# Supplementary material for: Current Perspectives on Idiopathic Intracranial Hypertension without Papilloedema
Source: Life (Basel). 2021 May 24;11(6):472. doi: 10.3390/life11060472 (PMC8225003; doi:10.3390/life11060472)
Supplement: Supplementary file 1 [file life-11-00472-s001.zip › life-1190041-supplementary.pdf]

S1: Search Strategy:

Database: Embase <1980 to 2020 Week 20>, Ovid MEDLINE(R) <1946 to May Week 2 2020>

Search Strategy:

1 Idiopathic intracranial hypertension without papilloedema.mp. [mp=ti, ab, hw, tn, ot, dm, mf, dv, kw, fx, dq, nm, kf, ox, px, rx, an, ui, sy] (16)

2 Idiopathic intracranial hypertension without papilledema.mp. [mp=ti, ab, hw, tn, ot, dm, mf, dv, kw, fx, dq, nm, kf, ox, px, rx, an, ui, sy] (70)

3 Idiopathic intracranial hypertension.mp. [mp=ti, ab, hw, tn, ot, dm, mf, dv, kw, fx, dq, nm, kf, ox, px, rx, an, ui, sy] (4682)

4 Pseudotumor cerebri without papilloedema.mp. [mp=ti, ab, hw, tn, ot, dm, mf, dv, kw, fx, dq, nm, kf, ox, px, rx, an, ui, sy] (0)

5 Pseudotumor cerebri without papilledema.mp. [mp=ti, ab, hw, tn, ot, dm, mf, dv, kw, fx, dq, nm, kf, ox, px, rx, an, ui, sy] (4)

6 Pseudotumor cerebri.mp. [mp=ti, ab, hw, tn, ot, dm, mf, dv, kw, fx, dq, nm, kf, ox, px, rx, an, ui, sy] (5861)

7 headache.mp. [mp=ti, ab, hw, tn, ot, dm, mf, dv, kw, fx, dq, nm, kf, ox, px, rx, an, ui, sy] (337196)

8 chronic headache.mp. [mp=ti, ab, hw, tn, ot, dm, mf, dv, kw, fx, dq, nm, kf, ox, px, rx, an, ui, sy] (3831)

9 refractory headache.mp. [mp=ti, ab, hw, tn, ot, dm, mf, dv, kw, fx, dq, nm, kf, ox, px, rx, an, ui, sy] (361)

10 migraine.mp. [mp=ti, ab, hw, tn, ot, dm, mf, dv, kw, fx, dq, nm, kf, ox, px, rx, an, ui, sy] (102058)

11 intracranial hypertension.mp. [mp=ti, ab, hw, tn, ot, dm, mf, dv, kw, fx, dq, nm, kf, ox, px, rx, an, ui, sy] (29897)

12 pulsatile tinnitus.mp. [mp=ti, ab, hw, tn, ot, dm, mf, dv, kw, fx, dq, nm, kf, ox, px, rx, an, ui, sy] (1735)

13 raised intracranial pressure.mp. [mp=ti, ab, hw, tn, ot, dm, mf, dv, kw, fx, dq, nm, kf, ox, px, rx, an, ui, sy] (4771)

14 7 and 12 and 13 (9)

15 3 and 7 (1868)

16 3 and 8 (63)

17 3 and 9 (16)

18 3 and 10 (326)

19 6 and 7 (1452)

20 6 and 8 (19)

21 6 and 9 (4)

22 6 and 10 (137)

23 1 or 2 (85)

24 4 or 5 (4)

25 15 or 16 or 17 or 18 or 23 (1947)

26 11 or 14 (29897)

27 25 and 26 (19)
